# Hepatoprotective Effect of *Oplopanax elatus* Nakai Adventitious Roots Extract by Regulating CYP450 and PPAR Signaling Pathway

**DOI:** 10.3389/fphar.2022.761618

**Published:** 2022-05-02

**Authors:** Xiao-Long Jiang, Pan-Yue Luo, Yan-Ying Zhou, Zhi-Hui Luo, Yue-Jun Hao, Ming-Zhi Fan, Xiao-Han Wu, Hao Gao, Hui-Chang Bi, Zhi-Bin Zhao, Mei-Lan Lian, Zhe-Xiong Lian

**Affiliations:** ^1^ Key Laboratory for Natural Resource of ChangBai Mountain & Functional Molecules, Ministry of Education, Yanbian University, Yanji, China; ^2^ School of Biomedical Sciences and Engineering, South China University of Technology, Guangzhou International Campus, Guangzhou, China; ^3^ School of Pharmaceutical Sciences, Sun Yat-Sen University, Guangzhou, China; ^4^ College of Pharmacy/Guangdong Province Key Laboratory of Pharmacodynamic Constituents of TCM and New Drugs Research, Institute of Traditional Chinese Medicine and Natural Products, Jinan University, Guangzhou, China; ^5^ Guangdong Provincial People’s Hospital, Guangdong Academy of Medical Sciences, Guangzhou, China

**Keywords:** *Oplopanax elatus* Nakai, adventitious roots, CYP450, neutrophil, PPAR, gut microbiota

## Abstract

*O. elatus* Nakai is a traditional medicine that has been confirmed to exert effective antioxidant and anti-inflammatory functions, and is used for the treatment of different disorders. However, its potential beneficial effects on drug induced hepatotoxicity and relevant molecular mechanisms remain unclear. This study investigated the protective effect and further elucidated the mechanisms of action of *O. elatus* on liver protection. *O. elatus* chlorogenic acids-enriched fraction (OEB), which included chlorogenic acid and isochlorogenic acid A, were identified by HPLC-MS/MS. OEB was administrated orally daily for seven consecutive days, followed by a single intraperitoneal injection of an overdose of APAP after the final OEB administration. The effects of OEB on immune cells in mice liver were analyzed using flow cytometry. APAP metabolite content in serum was detected using HPLC-MS/MS in order to investigate whether OEB affects CYP450 activities. The intestinal content samples were processed for 16 s microbiota sequencing. Results demonstrated that OEB decreased alanine aminotransferase, aspartate aminotransferase contents, affected the metabolism of APAP, and decreased the concentrates of APAP, APAP-CYS and APAP-NAC by inhibiting CYP2E1 and CYP3A11 activity. Furthermore, OEB pretreatment regulated lipid metabolism by affecting the peroxisome proliferator-activated receptors (PPAR) signaling pathway in mice and also increased the abundance of *Akkermansia* and *Parabacteroides*. This study indicated that OEB is a potential drug candidate for treating hepatotoxicity because of its ability to affect drug metabolism and regulate lipid metabolism.

## Introduction

Drug-induced liver injury (DILI) can be caused by prescription medicines, herbal products or dietary supplements and it is usually a result of mismedication or repeated medication in Europe and the United States (Bernal and Wendon, 2013). DILI induces many cellular responses, which include drug metabolism, oxidative stress, apoptosis/necrosis and immune response activation (Stephens et al., 2014). Therefore, DILI remains a challenge in clinic study and it is a main cause of acute liver failure in many countries. It is extremely important to find a therapeutic method or medicine to treat DILI.

Acetaminophen (APAP) is a non-steroidal analgesic that is commonly used in clinical practice and does not harm the liver at regular doses. However, APAP overdose is the leading cause of drug-induced liver failure, which makes it an ideal and practical model for investigating DILI (Shi et al., 2019). Although N-acetylcysteine (NAC) could treat APAP-induced liver injury (AILI) patients, the therapeutic window for NAC is limited due to the rapid onset of acute liver injury (Nakhaee et al., 2021). It has been shown that APAP is metabolized to the toxic strong electron-based metabolite N-Acetyl-p-benzoquinone imine (NAPQI) by cytochrome P450 enzymes (CYPs) such as CYP2E1, CYP1A2 and CYP3A4. NAPQI-detoxification is primarily accomplished by covalent binding with glutathione (GSH) to form APAP-glutathione, which is then rapidly hydrolyzed to form acetaminophen cysteine (APAP-CYS). APAP-CYS is then acetylated at the N-terminus to form 3-(N-Acetyl-L-cystein-S-yl) acetaminophen (APAP-NAC) (Newton et al., 1986). Only about 5%–10% of APAP is transformed to NAPQI at regular doses. However, during an overdose of APAP, excess NAPQI attaches covalently to functional proteins in hepatocytes with sulfhydryl groups, forming APAP-protein adducts that inactivate functional proteins, triggering hepatocyte necrosis and liver injury (Hairin et al., 2013).

A connection between metabolic and oxidative stress has been demonstrated in the early stages of injury. Overdose APAP induces oxidative stress in the mitochondria, resulting in cell damage and necrosis, as well as an inflammatory response following cell death (Cao et al., 2018). Neutrophils, a key component of the immune system, are linked to a variety of liver injury disease models. Neutrophils produce oxidants like H_2_O_2_ and release a number of functional enzymes to destroy dead and dying cells, thereby preparing the environment for wound healing and tissue regeneration. An overdose of APAP causes a large rise in neutrophil infiltration in the liver. When an inflammatory injury occurs, these cells flood into the liver, greatly raising the number of neutrophils (Kubes and Mehal, 2012; Szabo and Petrasek, 2015). Furthermore, previous studies show that APAP regulates lipid metabolism in the liver (Du et al., 2013). Peroxisome proliferator-activated receptors (PPAR) are mainly expressed in organs that are critical in fatty acid catabolism, suggesting that the most critical role of PPAR is to modulate hepatic fatty acid catabolism (Patterson et al., 2012; Li et al., 2017). Evidence suggests that altered gut microbiota may also impact the liver’s susceptibility to APAP hepatotoxicity and change its metabolism function. Besides, PPAR play a key role in autoimmune disease and chronic inflammation (Bae et al., 2020; Liu et al., 2020b).

Natural hepatoprotective agents from traditional Chinese medicines are gaining increased attraction in the treatment of AILI. *O. elatus* Nakai is an endangered plant found in Northeast China’s Changbai Mountains, with a lot of effective compounds, essential oils, total flavonoids, polysaccharides, saponin, and polyphenols. In traditional Chinese medicine, *O. elatus* has been used for treating neurasthenic, hypopiesis, schizophrenia, cardiovascular diseases, diabetes mellitus and rheumatism and it also possesses antifungal, fever-relieving, pain-relieving, and anti-aging activities (Shikov et al., 2014). However, the increasing scarcity of *O. elatus* wild resources and the difficulty of artificial cultivation which the reason why low resource utilization. To solve this problem, we use the bioreactor to culture the root of *O. elatus*. Bioreactor is a new plant tissue culture technology which has been successfully developed for culturing the *O. elatus* adventitious roots (ARs). Optimizing the culture conditions for *O. elatus* ARs in the bioreactor can largely increase its production (Jiang et al., 2021). Extracts from cultured *O. elatus* Ars have demonstrated excellent anti-inflammatory, antioxidant, and antibacterial activities in our previous study (Tian et al., 2019; Jin et al., 2020). However, the hepatoprotective effect of *O. elatus* ARs has not yet been investigated. According to its anti-inflammatory and antioxidant activities, we investigated OEB hepatoprotective effect in drug-induced liver injury. The aim of the present study was to determine whether OEB exhibited an inhibitory effect on CYP450 and whether OEB could regulate the lipid metabolism to decrease hepatotoxicity. In this study, we clarified the mechanism of hepatoprotective effect of the extract of *O. elatus* ARs.

## Materials and Methods

### Chemicals and Reagents

Acetaminophen (APAP) and 2, 2-diphenyl-1-picryhydrazyl radical (DPPH) were purchased from Sigma-Aldrich (St Louis, MI, United States). Chlorogenic acid, isochlorogenic acid A, 4-aminobenzoic acid (PABA), 4-methylpyrazole (4-ME), chlorzoxazone (CHL), nifedipine (NIF), NADPH were purchased from Yuanye biotechnology (Shanghai, China). 3-(N-Acetyl-L-cystein-S-yl) acetaminophen (APAP-NAC), acetaminophen cysteine (APAP-CYS), 6-OH-chlorzoxazone (6-OH-CHL), dehydro nifedipine (DNIF), ketoconazole (KET) was purchased from Toronto Research Chemicals (North York, ON, Canada). Loratadine (LOR) was purchased from Meilunbio (Dalian, China). HPLC grade methanol, acetonitrile, and formic acid were purchased from Merck (Darmstadt, Germany). Deionized water was generated using a Milli-Q system (Millipore, Bedford, MA, United States).

### Preparation of *O. elatus* AR’s Extract and Determination by LC-MS/MS

The *O. elatus* ARs were cultured in an air-lift balloon-type bioreactor using the method of our previous study (Jiang et al., 2015b; Han et al., 2019). The harvested ARs were dried at 45°C for 72 h after washing twice with tap water to remove the medium. To prepare the *O. elatus* ARs extract, the dried *O. elatus* ARs (500.0 g) were refluxed twice with 5 L 75% EtOH-H_2_O for 2 h each time. After filtration, the solution was removed under reduced pressure to yield a crude extract (OECE) (185.5 g∼ 37.1%). A portion of OECE (80.0 g) was passed through the HPD-722 macroporous resin column (7 cm × 120 cm) using a successive elution of EtOH-H_2_O (0:100, 50:50, 95:5, and *v/v*), yielding three fractions: OEA (38.6 g∼ 48.3%), OEB (25.9 g∼ 32.4%), and OEC (5.4 g∼ 6.8%) (Supplementary Figure S1A). Before LC-MS/MS analysis, 0.25 g of the extract was dissolved in 5 ml of 70% MeOH-H_2_O, sonicated for 30 min, centrifuged at 15,000 rpm for 10 min, and the supernatant was filtered through a 0.22 μM membrane to obtain the supernatant sample for LC-MS/MS analyzing.

The ESI-MS spectra were recorded on a Bruker amazon SL mass spectrometer (Bruker Daltonics Int., Boston, MA, United States). Analytical HPLC was performed on a Dionex HPLC system equipped with an Ultimate 3,000 pump, an Ultimate 3000 DAD, an Ultimate 3,000 column compartment, an Ultimate 3,000 autosampler (Thermo Fisher Scientific Inc., Sunnyvale, CA, United States), and an Alltech (Grace) 2000 ES ELSD (Alltech Co. Ltd., Portland, OR, United States) using a Cosmosil Packed C18 column (4.6 mm × 250 mm, 2.5 μm) (Nacalai Tesque Inc., Kyoto, Japan). Methanol (solvent A) and acidified water (0.1% formic acid, solvent B) were used as mobile phases with the following gradient: 0 min 10% A-35 min 73% A-45 min 90% A-55 min 100% A-65 min 100% A. The injection volume was 10.0 μl; the column temperature was set at 30°C; and the flow rate was 1.0 ml/min. The mass spectrum data were recorded in negative mode with a scan range of *m/z* 50–2,200.

### Animals and Experiments

Male C57BL/6 wild-type mice (6–8 weeks) were purchased from Hunan SJA Laboratory Animal Co., Ltd. (China). All the mice used in this study were bred under specific-pathogen-free conditions according to the guidelines for the Care and Use of Laboratory Animals of the South China University of Technology (Guangzhou, China).

Experimental design: an APAP-induced mouse model was established using the method referenced to Fan et al. (2015) with slight modification. Drug dosage needs to be calculated based on the specific weight of the mice. APAP (300 mg/kg) were dissolved saline at 45°C for 30 min, NAC (100 mg/kg) and OEB (50 and 100 mg/kg) were dissolved in saline and vortexed, all solution need filtered 0.45 μM membrane. All mice were randomly divided into six groups: 1) Normal control group (NT): Mice were given a definite volume of saline solution for seven consecutive days. 2) Model group (APAP): Mice were given a definite volume of saline solution (p. o.) for seven consecutive days, and treated with a single dose of APAP after final saline solution treatment. 3) NAC pretreated groups (NAC + APAP): Mice were given a definite volume of NAC (100 mg/kg) solution (p. o.) for seven consecutive days, and treated with a single dose of APAP after final NAC solution treatment. (4–5) OEB pretreated groups (OEB-L + APAP or OEB-H + APAP): Mice were given a definite volume of OEB (50 mg/kg or 100 mg/kg) solution (p. o.) for seven consecutive days, and treated with a single dose of APAP after final OEB solution treatment. 6) Only OEB treatment: Mice were given a definite volume of OEB (100 mg/kg) solution (p. o.) for seven consecutive days. Mice were sacrificed after APAP administrated 12 h and serum, liver tissues, and intestinal content samples were collected for further analysis.

### Serum Biochemical Levels Analysis

Enzymatic activities of aspartate aminotransferase (AST), alanine aminotransferase (ALT), total cholesterol (CHO) and triglycerides (TG) in serum were evaluated by spectrophotometer using diagnostic kits from Shanghai Kehua Bio-engineering Co., Ltd. (Shanghai, China).

### Liver Antioxidant Levels Analysis

Frozen liver samples were homogenized in pre-cooled PBS. The supernatants were collected after the homogenates were centrifuged at 3,000 × g, 4°C for 10 min. Superoxide dismutase (SOD), catalase (CAT), malondialdehyde (MDA) and glutathione (GSH) levels were measured with a spectrophotometer using the commercially available assay kits following manufacturer instructions (Solarbio, Beijing, China). The protein concentrations in the tissue homogenates were measured with Bradford protein assay using bovine serum albumin as the standard.

### Liver Histological Observation

Liver tissues were fixed in 10% formalin and embedded in paraffin for histological assessment. Samples were sectioned at 5 µm and stained with hematoxylin and eosin (H&E). The slides were examined under a light microscope with photo-micrographic attachment.

### LC–MS/MS Analysis of Acetaminophen Metabolites in Mice Serum

APAP metabolites were measured according to a previously reported method Dargue et al. (2020) with slight modifications. Serum sample preparation: 5 μl serum was spiked with 10 μl internal standard PABA solution (2 μg/ml) and 85 μl methanol, vortexed for 10 min, frozen at −20°C for 20 min, and then centrifuged at 15,000 rpm, 4°C for 15 min. 20 μl supernatant was diluted with 980 μl 0.1% formic acid water, vortexed and mixed thoroughly, centrifuged at 15,000 rpm and 4°C for 10 min, and then transferred to vials for analysis. APAP-CYS standard curves ranged from 34.375 to 30,000 ng/ml and APAP-NAC standard curves ranged from 15.625 to 2,000 ng/ml. The samples were prepared as described above, and the concentrations of the APAP metabolites were measured by the LCQUAN^TM^ below.

Chromatographic conditions: Liquid chromatography was performed on Ultimate 3000 UPLC system (Dionex Corporation, Sunnyvale, CA, United States). Hypurity C18 (2.1 mm × 150 mm, 5 μm) column (Thermo Fisher Scientific, Waltham, MA, United States) was used at 40°C; the mobile phase was methanol: 0.1% formic acid water (80:20, *v/v*) with equal gradient elution; the flow rate was 0.2 ml/min; the scan time was 5 min; the injection volume was 10 μl, and the injector temperature was 15°C.

Mass spectrometry conditions: Mass Spectrometry detection was carried out with triple quadrupole mass spectrometer (TSQ Quantum Access, Thermo Fisher Scientific, Waltham, MA, United States). The ion source was an electrospray ion source with a positive spray voltage of 2,500 V and a capillary temperature of 350°C. Detection of the analyte ions was performed under the selective reaction monitoring (SRM) mode. The transition monitored for the analytes were APAP-NAC, [M + H]^+^, *m/z* 312.90→207.80; APAP-CYS, [M + Na]^+^, *m/z* 271.00→139.90; PABA, [M + H]^+^, *m/z* 138.10→120.00, respectively.

### Liver Microsomes Extraction and Sample Treatment

Fresh mice liver samples were rinsed with pre-cooled sucrose solution at 4°C. Two times the volume of liver weight of sucrose solution was added to the liver samples. The solution was manually homogenized on ice, and centrifuged at 16,000 × *g*, at 4°C for 20 min. The collected supernatants were placed in ultra-high speed centrifuge tubes, and centrifuged at 100,000 × *g*, at 4°C for 60 min. Supernatants were discarded and the collected precipitate was washed with potassium pyrophosphate solution and centrifuged again at 100,000 × *g*, at 4°C for 60 min. The supernatant was discarded, and the microsomes were quantified by resuspension in Tris-HCl buffer.

Hepatic microsomes incubation and sample treatment: Mixed mouse liver microsomes and probe substrates (CHL and NIF), KET and 4-ME were added to the positive control group; methanol was added to the blank group, and OEB solutions (OEB-L 2.5 μg/ml, OEB-M 5.0 μg/ml, OEB-H 10.0 μg/ml) were added to the drug administration group. The system was pre-incubated at 37°C in a metal bath; the reaction was initiated by adding NADPH and incubating for 20 min at 37°C in a water bath; and terminated by adding cold ethyl glacial acetate. After adding 10 μl internal standard LOR solution (2 μg/ml) to the system, it was vortexed for 2 min, rested for 10 min and centrifuged at 3,500 rpm for 10 min. The supernatant was transferred and the organic solvent was evaporated. Before injection, 200 μl of 80% methanol water was added, vortex shaken for 1 min, and centrifuged at 16,000 rpm for 5 min to produce the sample.

### Measurement of Liver CYP450 Enzyme Activity

Liver CYP450 enzyme activity was measured according to a previously reported method (Jiang et al., 2015a) with slight modifications. Chromatographic conditions: Waters’ Xetrra C18 (2.1 mm × 100 mm, 5 μm) column was used at 40°C; the mobile phase was methanol: 0.1% formic acid in water (70:30, *v/v*) with equal gradient elution; the flow rate was 0.3 ml/min; the analysis time was 3.5 min; the injection volume was 10 μl; and the injector temperature was 15°C.

Mass spectrometry conditions: The ion source was an electrospray ion source with a positive spray voltage of 3,500 V, a negative spray voltage of 2,500 V, and a capillary temperature of 350°C. Detection of the analyte ions was performed under SRM mode.

The transitions monitored for the analytes were 6-OH-CHL, [M-H]^-^, *m/z* 184.107→120.20; DNIF, [M + H]^+^, *m/z* 345.132→284.107; LOR, [M + H]^+^, *m/z* 382.00→266.00, respectively. The concentration ranges included 6-OH-CHL from 0.1 to 6.4 μmol/L, and DNIF from 0.125 to 4 μmol/L. All the samples were measured in the HPLC-MS/MS system to determine the content of the substrate metabolites in the liver microsomes incubated system and to calculate CYP450 enzyme activity.

### Flow Cytometry

Liver cell suspensions were blocked with anti-mouse CD16/32 before surface staining. The cell suspensions were incubated with fluorochrome-conjugated monoclonal antibodies in PBS with 0.2% bovine serum albumin at 4°C for 20 min. The monoclonal antibodies included anti-Gr-1 (FITC and RB6-8C5), anti-CD11c (PerCP-Cy5.5 and N418), anti-KLRG1 (PE and 2F1/KLRG1), anti-NK1.1 (PE-Cy7 and PK136), anti-CD19 (PE-Cy5 and 6D5), anti-CD44 (Alexa647 and IM7), anti-CD45.2 (APC/Cy7 and 104), anti-I-A/I-E (Alexa700 and M5/114.15.2), anti-CD11b (V500 and M1/70), anti-CD3 (BV421 and 17A2), anti-CD4 (BUV563, GK1.5, BD Biosciences), anti-CD8a (BV711 and 53–6.7), anti-CD49a (BUV395, HA31/8, BD Biosciences), anti-CD62L (BUV737, 145-2C11, BD Biosciences), and anti-CD69 (BV605, H1.2F3) according the reference with slight modifications (Huang et al., 2021). All fluorochrome-conjugated antibodies, unless otherwise noted, were purchased from Bio Legend (San Diego, CA, United States). Data were acquired with a BD LSRFortessa flow cytometer, then analyzed with the Flowjo software. T-distributed stochastic neighbor embedding (t-SNE) analysis was used to profile liver infiltrating lymphocytes subsets in R language. The expression matrixes of CD45^+^ immune cells from APAP group (*n* = 3) and OEB + APAP group (*n* = 3) mice were exported for analysis. 3,000 cells were randomly selected from each sample, then merged into one single matrix containing 15 channels. Normalized expression matrix by RunTSNE function in Seurat package (version 3.0.1).

### RNA and 16s Sequence Analysis

Liver and intestinal samples were collected from the APAP group mice and OEB + APAP group mice, quick-frozen in sterile tubes with liquid nitrogen and stored at −80°C until further use. Liver samples were processed for RNA sequencing by Novegene Bio-Pharm Technology Co., Ltd (Beijing, China). The data were analyzed on R language. The intestinal content samples were processed for 16s microbiota sequencing by Majorbio Bio-Pharm Technology Co., Ltd (Shanghai, China). The data were analyzed on the free online platform of Majorbio Cloud Platform.

### Statistical Analysis

All values are expressed as means ± standard error of the mean. Data were analyzed by one-way analysis of variance (ANOVA) using GraphPad Prism 8.0 (San Diego, CA, United Statess), and then differences among means were determined using Dunnett’s multiple comparisons test. Statistically significant difference was set at *p* < 0.05.

## Results

### Inhibition of APAP-Induced Hepatotoxicity by OEB

The extraction process of *O. elatus* ARs is as the flow chart is shown in Supplementary Figure S1A, and we got OECE, OEA, OEB, and OEC, respectively. Furthermore, we found clearly chlorogenic acids-enriched compounds (chlorogenic acid and isochlorogenic acid A) in the OEB fraction (Supplementary Figures S1B–D).

**FIGURE 1:**
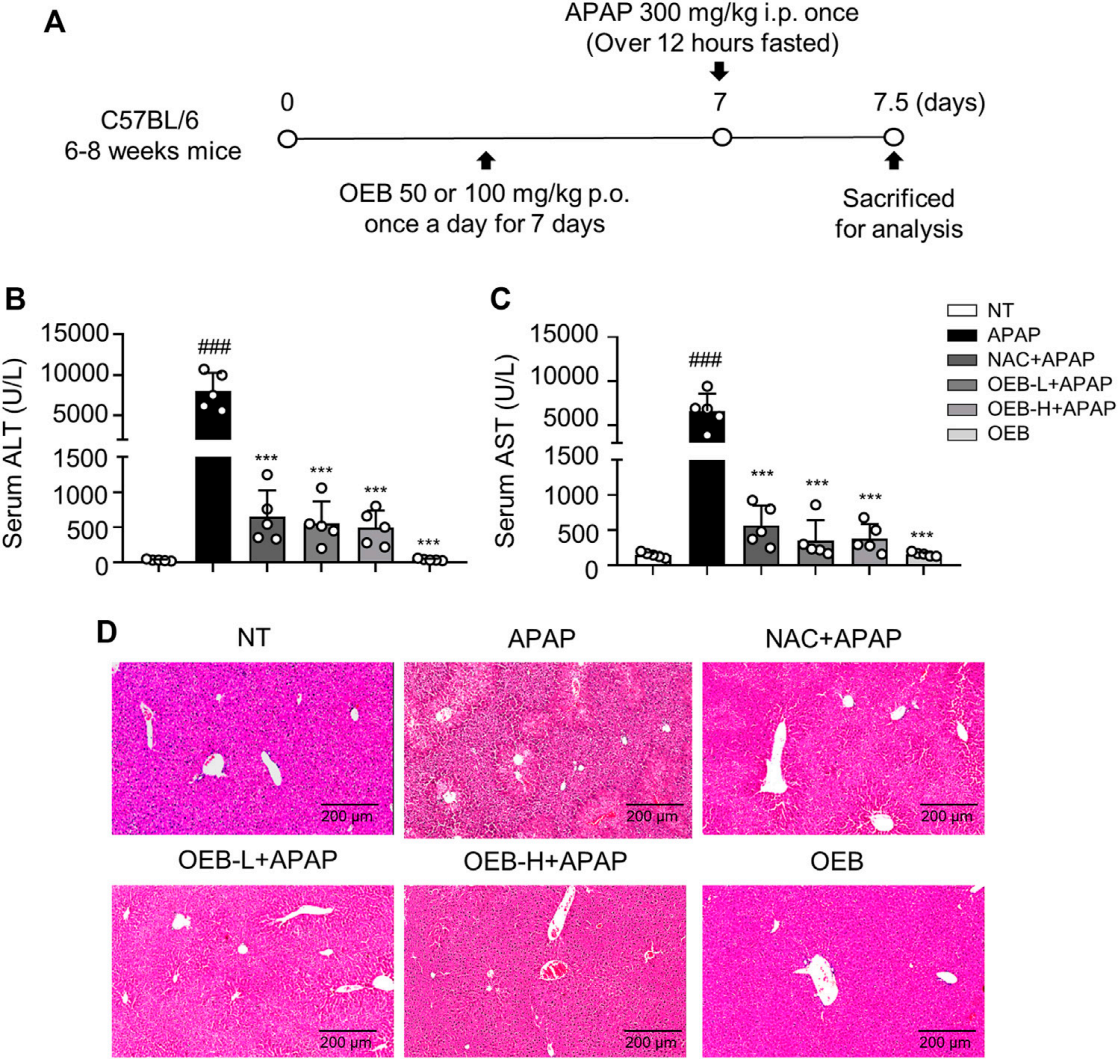
Protective effect of OEB in APAP-induced hepatotoxicity *in vivo*. **(A)** Mice were pretreated with 50 mg/kg OEB by intragastric administration for 7 days and administered a single dose of 300 mg/kg APAP by intraperitoneal injection and euthanized after 12 h. Hepatotoxicity was analyzed by measuring serum **(B)** ALT and **(C)** AST levels (*n* = 5). **(D)** Hematoxylin and eosin-stained liver sections. ***p < 0.001 versus APAP-treated group, ^###^ *p* < 0.001 versus NT group.

We then designed experiments to determine whether OEB has hepatoprotective activity (Figure 1A). H&E staining of liver sections showed that the area of necrosis was significantly greater in the APAP group as compared to that in the normal group (NT), while the degree of injury was significantly lower in the OEB pretreated group than that in the APAP group (Figure 1D). Serum levels of ALT and AST were elevated after APAP treatment compared to those of the NT group, which indicated that hepatocellular damage induced by APAP was successfully established. Supplementation with 50 mg/kg OEB for 7 days significantly inhibited the increase in ALT and AST levels after exposure to APAP treatment (Figure 1B,C). We then investigated the OEB’s toxicity *in vivo*, and found that OEB has no effect on liver damage in mice (Figure 1D). These results indicated that OEB had hepatoprotective activity and reduced APAP-induced liver injury (AILI) in mice.

### Inhibition of Cytochrome P450 Enzymes Activity and Acetaminophen Metabolism in APAP-Induced Liver Injury by OEB

**FIGURE 2:**
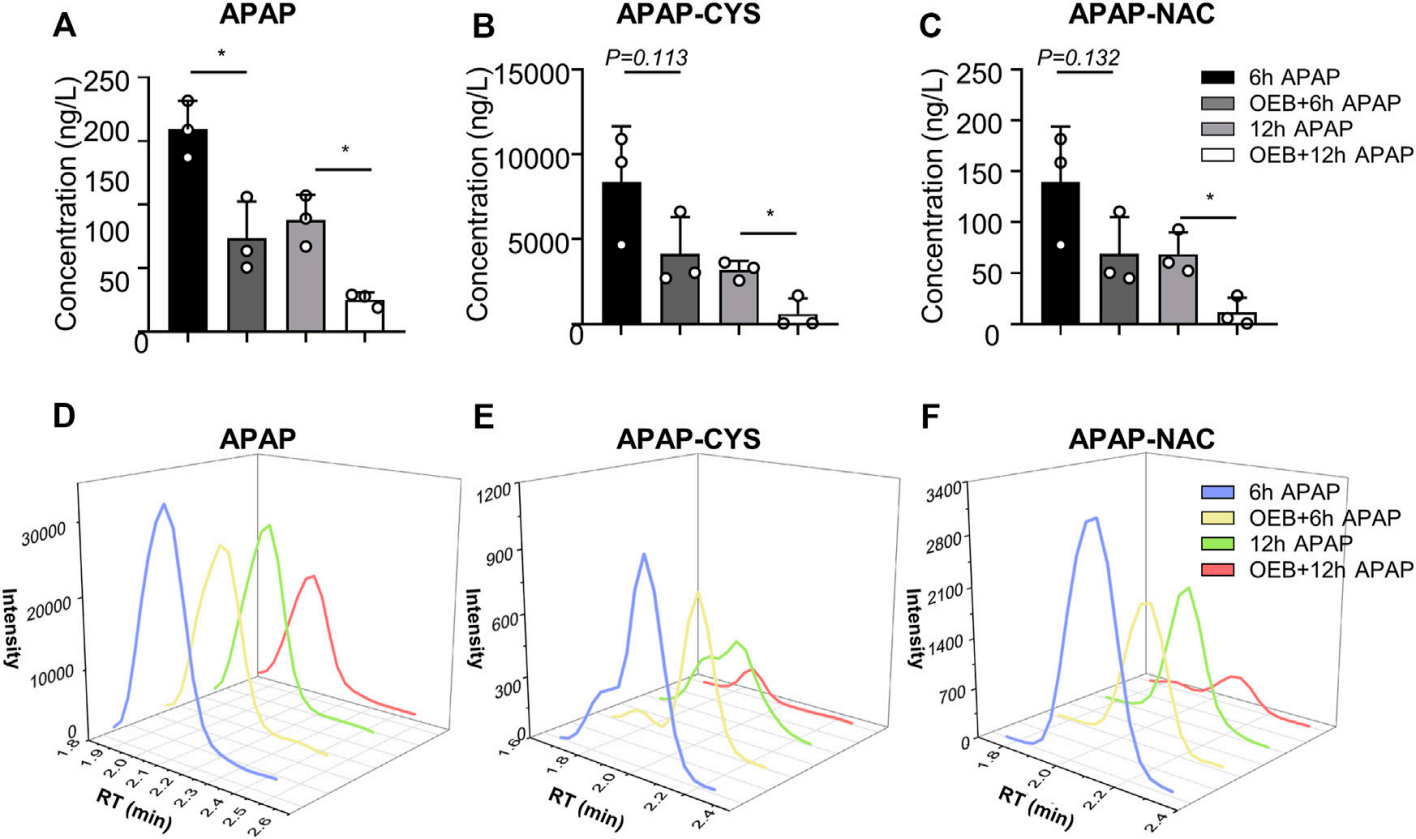
OEB reduced APAP-induced hepatotoxicity through regulating APAP-metabolism. **(A)** APAP content, **(B)** APAP-CYS content and **(C)** APAP-NAC content in mice serum, were measured by HPLC-MS/MS after administering APAP 6 h and 12 h (*n* = 3). Intensities of **(D)** APAP, **(E)** APAP-CYS and **(F)** APAP-NAC were analyzed by HPLC-MS/MS. *p < 0.05 versus APAP-treated group.

Overdoses of APAP induced liver injury is mediated by toxic APAP adducts first produced by CYPs. Therefore, we investigated whether OEB interferes with the metabolism and detoxification of APAP. The concentrations of APAP and APAP metabolites (APAP-CYS and APAP-NAC) in mice serum were measured using the established HPLC-MS/MS method. Compared to the APAP treatment group, the contents and intensities of APAP, APAP-CYS and APAP-NAC **(** Figure 2) of OEB pretreatment group were significantly decreased (*p < 0.05).

**FIGURE 3:**
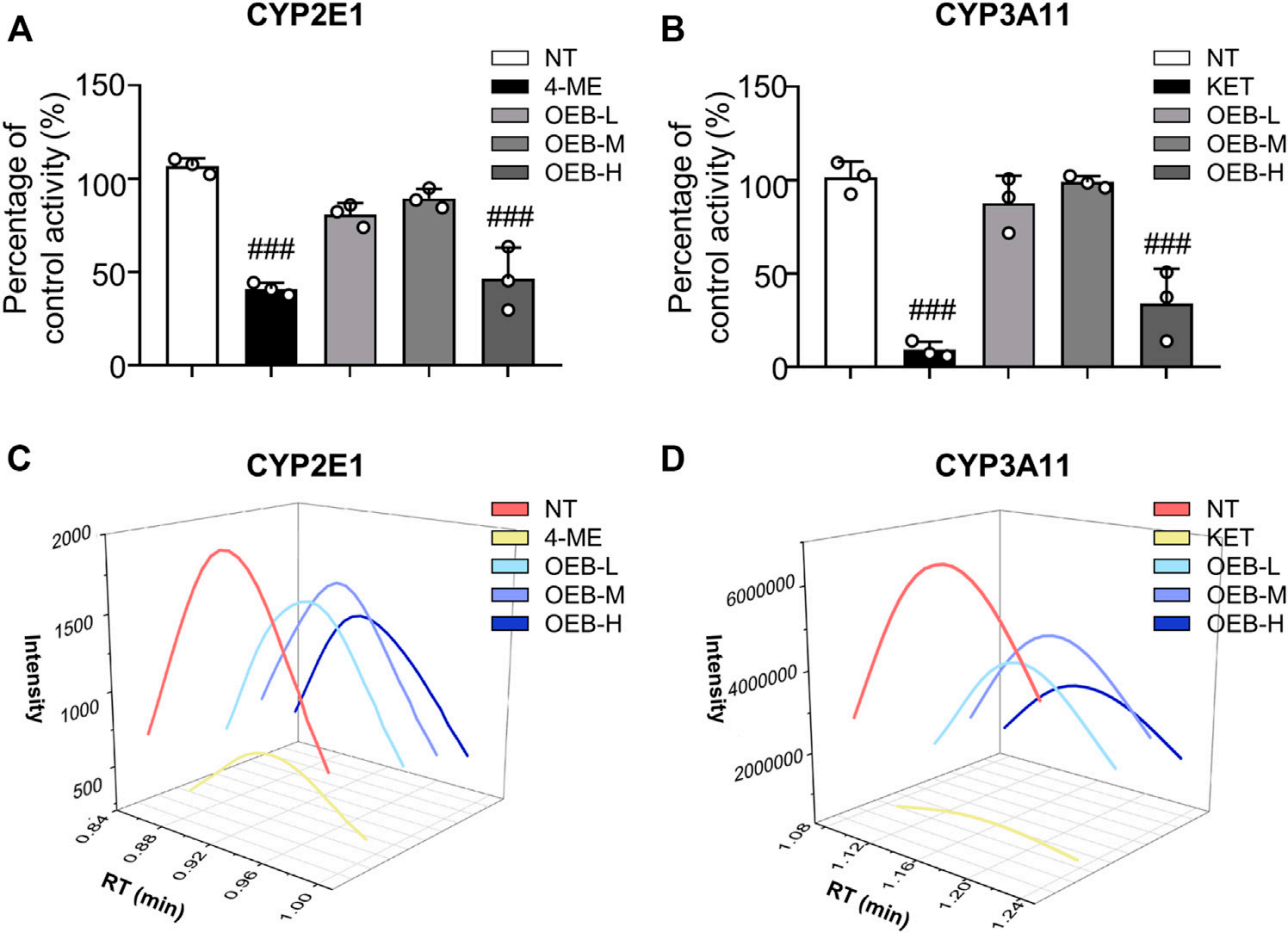
OEB reduced APAP-induced hepatotoxicity through regulating cytochrome P450 activity. Drug metabolizing enzymes **(A)** CYP2E1 and **(B)** CYP3A11 activities were measured in hepatic microsomes by HPLC-MS/MS (*n* = 3). Intensities of **(C)** CYP2E1 and **(D)** CYP3A11 were analyzed by HPLC-MS/MS. ^*###*^ *p* < 0.001 versus NT group.

**FIGURE 4:**
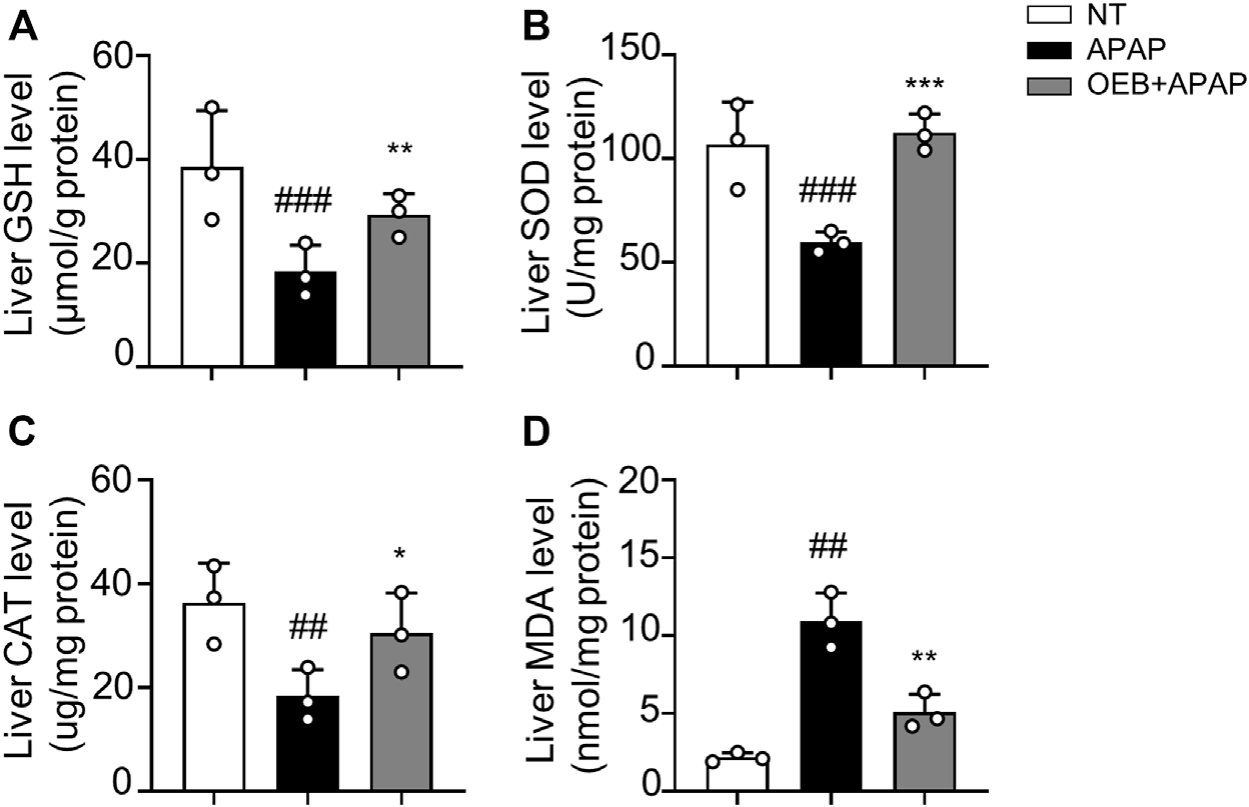
OEB reduced APAP-induced hepatotoxicity *via* regulating oxidative enzyme activities. **(A)** Glutathione (GSH), **(B)** superoxide dismutase (SOD), **(C)** catalase (CAT) and **(D)** malondialdehyde (MDA) levels in the liver tissue were measured. (*n* = 3). Data are representative of three independent experiments with similar results. *p < 0.05, **p < 0.01, ***p < 0.001 versus APAP-treated group. ^*##*^ *p* < 0.01, ^###^ *p* < 0.001 versus NT group.

To further evaluate the effect of OEB on CYP2E1 and CYP3A11 activity, liver microsomes incubation system with OEB and CYPs substrates was performed *in vitro*. The reactions were carried out using CYP2E1 and CYP3A11 substrates (CHL and NIF), CYP2E1 and CYP3A11 inhibitors (4-ME and KET), and OEB at various concentrations (2.5, 5.0, and 10.0 μg/ml). The relative enzyme activities were detected using the HPLC-MS/MS method. As shown in Figures 3A–D, compared to the control group, the OEB pretreatment group showed significant (***p < 0.001) inhibition of CYP2E1 and CYP3A11 activity at 10 μg/ml. We also found the same phenomenon *in vivo*, as OEB significantly decreased (***p < 0.001) the expression of CYP2E1 and CYP3A11 (Supplementary Figures S2A,B). These results demonstrated that the activities of CYP2E1 and CYP3A11 were affected by OEB during the metabolic process of APAP in the liver. These results suggested that OEB reduced APAP-induced hepatotoxicity by inhibiting CYPs activity and decreasing the APAP metabolic activation. Our results also indicated that OEB inhibited the depletion of GSH and restored hepatic SOD and CAT activities caused by APAP **(** Figures 4A–C **)**. In addition, OEB could also block the APAP-induced increase of MDA levels in the liver (Figure 4D). These results demonstrated that OEB might reduce the effects of APAP induced oxidative stress.

### Inhibition of Neutrophils in APAP-Induced Liver Injury by OEB

**FIGURE 5:**
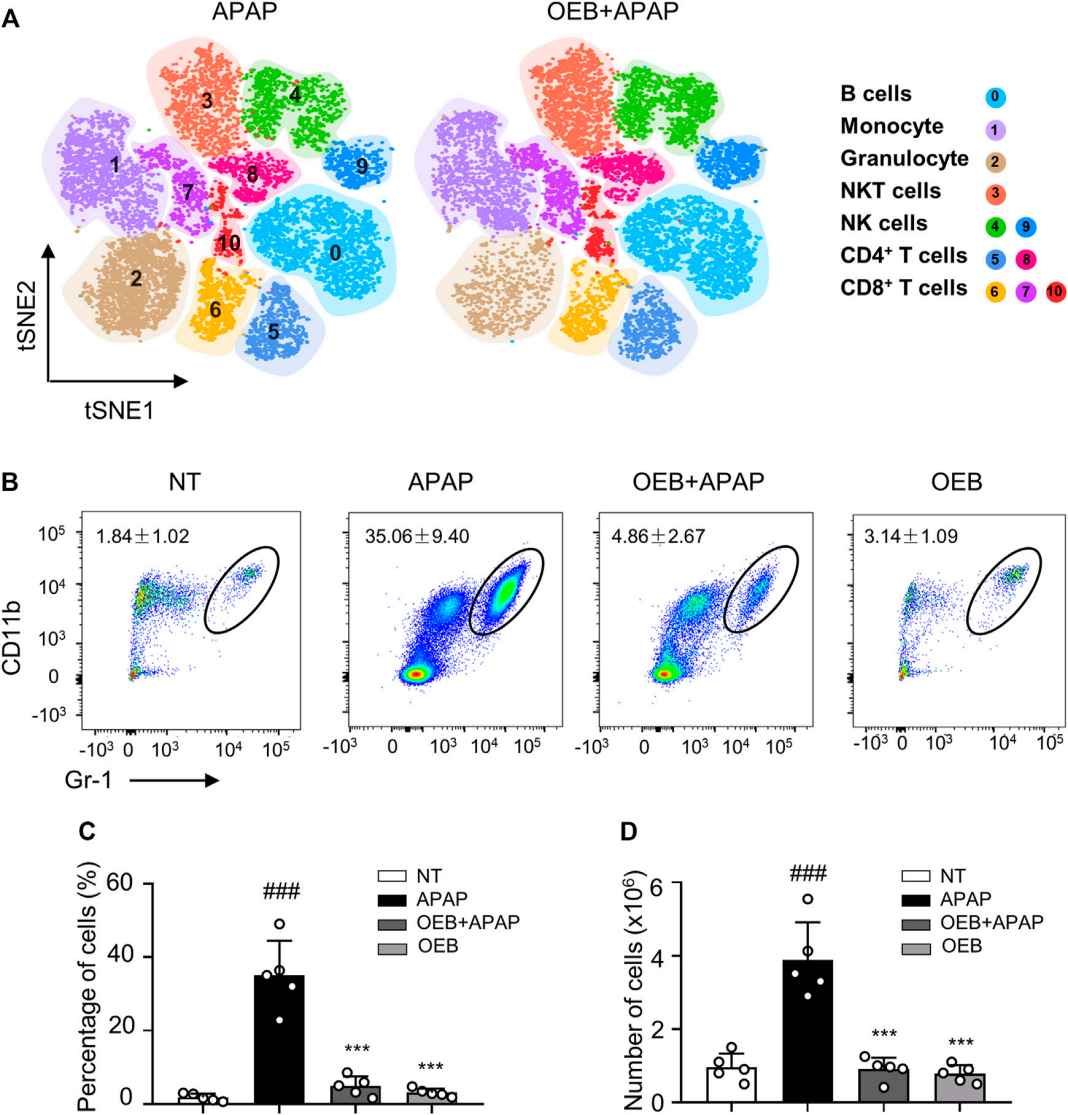
OEB reduced APAP-induced hepatotoxicity by affecting immune cells in the liver. **(A)** Merged t-SNE plots of flow cytometry data from liver samples of APAP (*n* = 3) and OEB + APAP (*n* = 3) groups. **(B)** Representative flow cytometry data for hepatic CD11b^+^ Gr-1 ^high^ neutrophil infiltration in the liver of APAP and OEB + APAP groups. The total neutrophil (CD11b^+^ Gr-1 ^high^ cells) **(C)** percentages and **(D)** numbers in the liver of APAP and OEB + APAP groups (*n* = 5). Data are representative of three independent experiments with similar results. ***p < 0.001 versus APAP-treated group. ^*###*^ *p* < 0.001 versus NT group.

Most study found overdose APAP may impact hepatotoxicity by affecting immune cells (Jaeschke et al., 2011). Our findings also suggest that OEB pretreatment affects immune cells in APAP-induced hepatotoxicity, especially neutrophils. We performed a t-distributed stochastic neighbor embedding (t-SNE) analysis using the flow cytometry data, and identified 11 immune cell subtypes in the liver based on the 15 cell surface markers, including one cluster of B cells (cluster 0), one cluster of monocyte cells (cluster 1), one cluster of granulocyte cells (cluster 2), one cluster of NKT cells (cluster 3), two clusters of NK cells (clusters 4 and 9), two clusters of CD4^+^ T cells (clusters 5 and 8), and three clusters of CD8^+^ T cells (clusters 6, 7 and 10) (Figure 5A). Compared to the control group, we observed the neutrophils percentage of the APAP group (35.06% ± 9.40%) significantly increased (^*###*^ *p* < 0.001). However, the neutrophils percentage of the OEB pretreatment (4.86% ± 2.67%) and OEB-only treatment groups (3.14% ± 1.09%) were both significantly decreased (***p < 0.001) compared to APAP group. (Figures 5B,C). The neutrophils number of the APAP group (3.88 ± 1.03 × 10^6^) significantly increased (###*p* < 0.001). However, the neutrophils number of the OEB pretreatment (0.91 ± 0.31 × 10^6^) was significantly decreased (***p < 0.001) compared to APAP group (Figure 5D). These findings indicate that OEB significantly affected immune cells and decreased the APAP-induced neutrophil infiltration.

### Regulation of Peroxisome Proliferator-Activated Receptors and Lipid Metabolism Signaling Pathway by OEB

**FIGURE 6:**
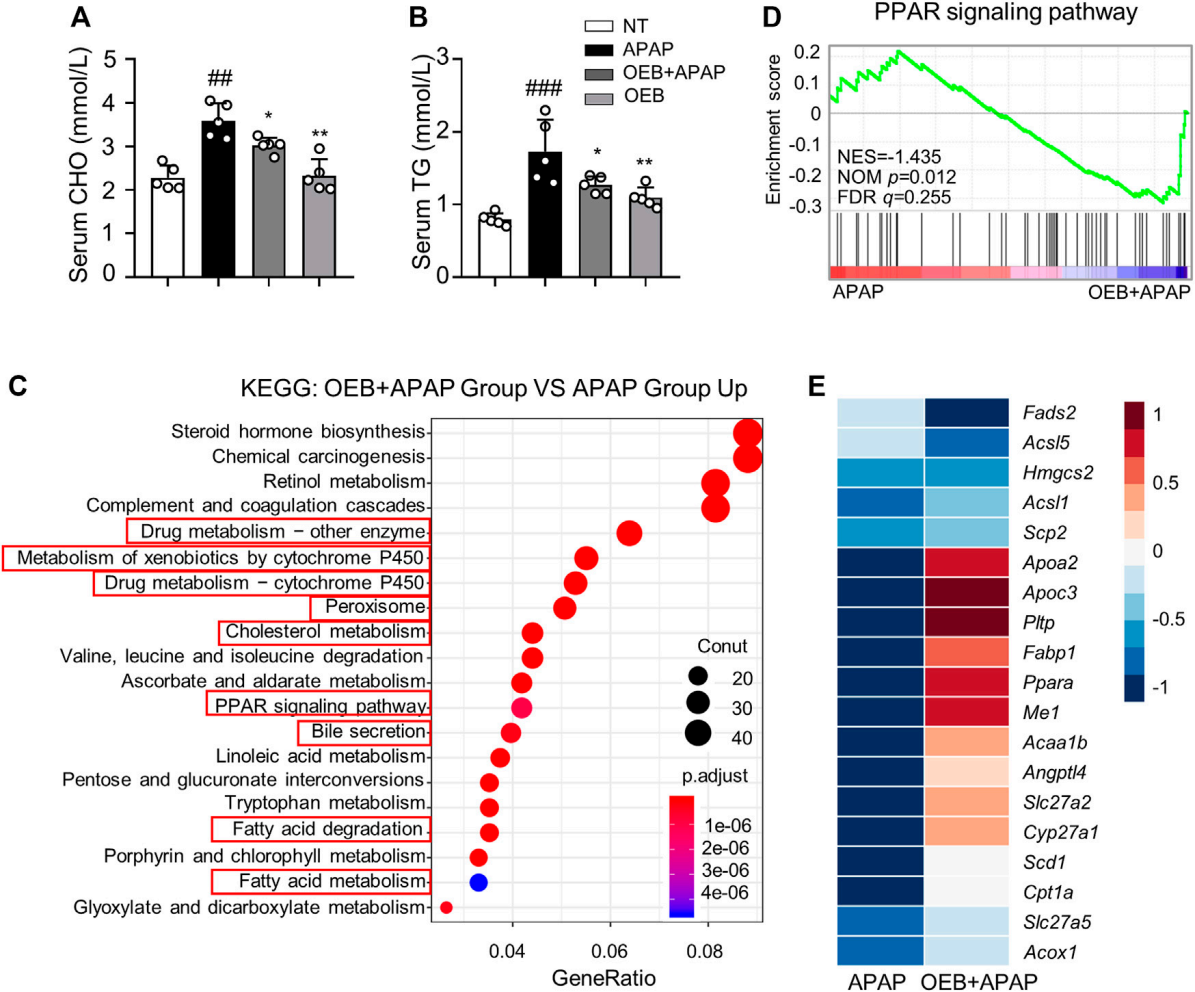
RNA-Seq analyses revealed significant difference in PPAR lipid metabolism between the presence or absence OEB pretreated in the APAP treated groups. Hepatotoxicity was analyzed by measuring serum **(A)** CHO and **(B)** TG levels (*n* = 5). **(C)** KEGG pathway enrichment analyses of the OEB + APAP (*n* = 3) and APAP (*n* = 3) groups. **(D)** GSEA pathway enrichment analysis of pathways related to PPAR signaling pathway. **(E)** Heatmap of PPAR-related gene expression profiles based on the GSEA results. *p < 0.05, **p < 0.01 versus APAP-treated group. ^*##*^ *p* < 0.01, ^*###*^ *p* < 0.001 versus NT group.

APAP can affect the lipid metabolism process in AILI, thereby we investigated the CHO and TG levels in serum. Our results found that OEB pretreatment significantly (*p < 0.05) decreased TG (1.27 ± 0.12 mmol/L) and CHO (3.03 ± 0.18 mmol/L) contents in mice serum compared to the APAP group (1.73 ± 0.44 mmol/L and 3.60 ± 0.40 mmol/L, respectively) (Figures 6A,B). To further explore the mechanism by which OEB protects against APAP-induced hepatotoxicity, we analyzed the liver samples from mice in the APAP group and OEB pretreatment groups by RNA-sequencing. RNA-Seq analysis showed that OEB pretreatment affected various signaling pathways related to drug metabolism-cytochrome P450 and other enzymes, cholesterol metabolism, linoleic acid metabolism, lipid metabolism, fatty acid metabolism, peroxisome and the PPAR signaling pathway (Figure 6C). GSEA systematically revealed that cellular signaling pathways related to PPAR were enriched and significantly downregulated by OEB pretreatment (Figure 6D). Furthermore, the heatmap based on GSEA revealed that the expression of hepatic genes related to PPAR and cholesterol metabolism signaling were significantly upregulated by OEB pretreatment in mice administered with APAP (Figure 6E). We also investigated the mRNA expression of PPAR related genes (*Pparα, Fabp1, Acaa1b*, and *Apoc3*) (Supplementary Figure S3), We found APAP treatment inhibited *Pparα, Fabp1, Acaa1b*, and *Apoc3* expression levels. However, after OEB pretreatment, the expression of *Pparα* (*p < 0.05), *Fabp1* (*p < 0.05), *Acaa1b* (**p < 0.01), and *Apoc3* (**p < 0.01) significantly increased. These results indicate OEB offers hepatoprotection by regulating PPAR signaling and lipid metabolism.

### Regulation of Gut Microbiota in APAP-Induced Liver Injury by OEB

**FIGURE 7:**
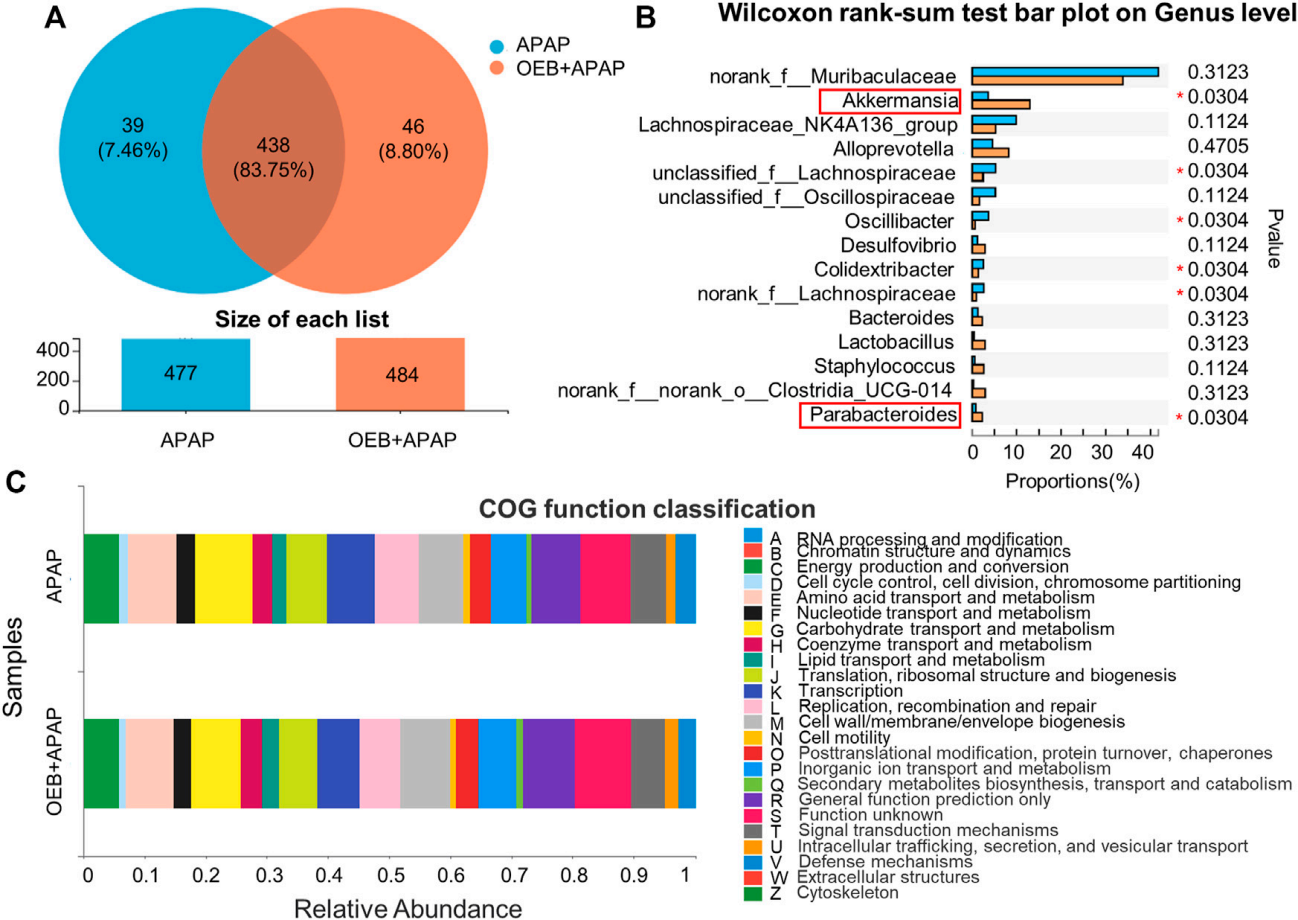
16s-Seq analyses revealed significant difference in gut microbiota between the presence and absence of OEB pretreatment in the APAP treated groups. **(A)** Venn diagram showing the number of specific commensal gut microbiomes and the overlap between APAP (*n* = 4) and OEB + APAP (*n* = 4) groups. **(B)** Wilcoxon rank-sum test bar plot revealed differentially enriched microbiota between APAP (*n* = 4) and OEB + APAP (*n* = 4) groups on genus level. **(C)** COG function classification analysis revealed differentially enriched bacterial functions associated between APAP (*n* = 4) and OEB + APAP (*n* = 4) groups. *p < 0.05 versus APAP-treated group.

To determine if OEB affected the role of the gut microbiota in APAP-induced liver injury, we investigated the profile of gut microbiota based on gut microbiomes samples from the mice in the APAP group and the OEB pretreatment group by 16s sequencing. Venn diagrams showed that mice in the APAP group had 39 specific microbiotas at the genus level and mice in the OEB pretreatment group had 46 specific microbiotas (Figure 7A). With the Wilcoxon rank-sum test bar plot analysis, we further found that the percentage of community abundance of *Lachnospiraceae*, *Oscillibacter* and *Colidextribacter* was lower in the OEB pretreatment group compared with that in the APAP group. However, the percentage of *Akkermansia* (13.02% ± 5.53%) and *Parabacteroides* (2.27% ± 1.56%) in OEB + APAP group was significantly higher (*p < 0.05) than APAP group (3.55% ± 1.81% and 0.80% ± 0.12%, respectively) on the genus level (Figure 7B). It is known that gut microbiota can regulate metabolic functions in acute liver injury. We then investigated whether OEB affected the functional potential of gut microbiota in two groups. With COG function classification analysis, lipid transport and metabolism were found as well (Figure 7C). Overall, we observed OEB pretreatment affected lipid metabolism by increasing the abundance of *Akkermansia* and *Parabacteroides*.

## Discussion

APAP is normally metabolized in the liver, and its hepatotoxicity is inextricably related to its metabolic role in the liver. APAP reaches the liver and interacts with glucuronosyltransferase and sulfotransferase to form non-toxic metabolites (APAP-glucuronide and APAP-sulfate), which are excreted in urine at regular doses. APAP can also be metabolized by cytochrome P450 enzymes (CYP2E1, CYP1A2, CYP3A4, etc.) which are covalently bound to GSH and thus excreted in the early stages of APAP metabolism to toxic metabolites N-Acetyl-p-benzoquinoneimine (NAPQI) (Przybya et al., 2020). Under regular doses, only 5 %–10% of APAP is biotransformed to NAPQI, which is detoxified mainly by covalent binding to GSH to generate APAP-glutathione. APAP-glutathione is rapidly hydrolyzed to APAP-CYS, which followed by N-terminal acetylation generates APAP-NAC. When APAP levels are high, hepatocytes produce too much NAPQI, which continuously binds to GSH, depleting GSH and disrupting redox homeostasis, resulting in oxidative stress (Du et al., 2013). Excess NAPQI binds to mitochondrial proteins, resulting in mitochondrial dysfunction, permeability changes, apoptosis, and necrosis. As a result, acetaminophen’s hepatotoxicity is attributed to oxidative stress, APAP-metabolism and mitochondrial dysfunction (Hanawa et al., 2008; Hairin et al., 2013). NAPQI is the main active substance that causes liver injury. However, its levels cannot be measured directly. The current assay used is to detect APAP-CYS and APAP-NAC produced by enzymatic digestion of APAP protein adducts, and use them to evaluate the NAPQI content *in vivo*. The protective effect of the adventitious root of *O. elatus* chlorogenic acids-enriched extract (OEB) against AILI was investigated for the first time in the present research. Our data found that OEB decreased ALT, AST, CHO, TG levels and APAP, APAP-CYS, APAP-NAC concentrations in serum. According to the mechanism of APAP metabolism, APAP content was decreased with time dependence in serum in the early stage (Lickteig et al., 2007). Except CYP450 action, most APAP were transformed into APAP-sulfonate and APAP-glutathione by glucuronosyltransferase and sulfotransferase, then excreted in urine. (Ben-Shachar et al., 2012). Thereby, OEB decreased APAP concentration in serum by glucuronosyltransferase and sulfotransferase (Supplementary Figure S2C). Furthermore, 10 μg/ml OEB affected CYP2E1 and CYP3A11 activities *in vitro* (Figure 3), thereby affecting APAP-metabolism.

Overdoses of APAP increase hepatotoxicity by inducing oxidative stress, and hepatocytes undergo apoptosis and necrosis as a result of this rapid onset of oxidative stress. Several studies have shown that natural medicine has antioxidative effect through reducing oxidative stress promotes hepatocyte suppression and hepatic function safety (Ahmad et al., 2012; Naguib et al., 2014; Hamza et al., 2015). Studies have shown that overdosing APAP produces NAPQI under CYP450 and induces mitochondria oxidative stress. Anti-oxidative enzymes (GSH, SOD, CAT, et al.) also defend the liver against oxidative stress in this process (Zhao et al., 2018). By detecting MDA, GSH, SOD and CAT levels, our results demonstrated that OEB pretreatment increased the activity of anti-oxidative enzymes and inhibited the production of hepatic oxidative stress in AILI (Figure 4).

Immune cell recruitment and infiltration causes extreme innate immune responses and sterile inflammation, all of which are necessary for the progression of mild liver injury to late liver failure (Jaeschke et al., 2011). APAP induces oxidative stress in the mitochondria, resulting in cell damage and necrosis, as well as an inflammatory response following cell death. Damage-Associated Molecular Patterns (DAMPs) release large amounts of cellular inflammatory factors and chemokines, which stimulate immune cells and affect inflammatory responses when they bind to their corresponding receptors (Martin-Murphy et al., 2010). DAMPs activate Kupffer cells, which release chemokines and chemokines receptors (CXCL1, CXCL2, and CXCL8), affect neutrophil infiltration, thereby increasing ROS levels and sterile inflammation. In the early stages of APAP overdose, neutrophils activate an injury process to hepatocyte in AILI (Wen et al., 2021). According to our current data, pretreatment with OEB regulated NK, NKT (data not shown), and significantly reduced neutrophil infiltration in the liver. However, studies have shown similar findings indicating that neutrophil activation may be a critical event for host defense or injury resolution following APAP overdose, but is not a contributing factor to APAP-induced injury (Liu et al., 2020a) (Williams et al., 2014). As a result, neutrophils’ role in the AILI model is convoluted, and the effect of OEB on neutrophils requires more research.

APAP-induced hepatotoxicity activated lipid metabolism in liver. Peroxisome proliferator-activated receptors (PPAR), a member of the nuclear receptor superfamily, control the expression of a battery of genes involved in lipid homeostasis, including those encoding peroxisome and mitochondrial enzymes that carry out fatty acid catabolism (Chinetti et al., 2000). Consequently, increased PPAR activity during accelerated fatty acid catabolism is associated with increased expression of free-radical scavengers such as catalase that may serve to reduce mitochondrial ROS levels. Both direct and indirect effects suggest that PPAR may serve a protective role in combatting the deleterious side effects of fatty acid catabolism, thus preserving mitochondrial function (Patterson et al., 2012). It has been reported that APAP affects the lipid metabolism (Gong et al., 2018), producing ROS to increase oxidant stress, especially the PPAR signaling pathway (Li et al., 2017). High levels of ROS in the cytoplasm will disrupt the mitochondrial membrane and spill over into the cytoplasm, causing apoptosis. Overdoses of APAP deplete GSH while also forming adducts with intercellular proteins, especially those covalently bound to mitochondrial proteins, causing respiratory chain dysfunction and promoting massive ROS development (Du et al., 2013). Our study showed that OEB + APAP group significantly (*p < 0.05) increased TG and CHO contents in mice serum compared to the APAP group (Figures 6A,B). TG and CHO contents could express the lipid metabolism level variation in non-alcoholic fatty liver disease, alcoholic fatty liver, drug induced liver injury and other liver disease. Therefore, OEB might regulate the lipid metabolism in APAP-induced hepatotoxicity process. Furthermore, we found that OEB regulated the lipid metabolism level in RNA sequencing data (Figure 6C) and OEB inhibited the PPAR signaling pathway related to gene expression (Supplementary Figure S3). These results showed that OEB had hepatoprotective effect through PPAR signaling pathway regulating lipid metabolism.

Susceptibility to APAP-induced hepatotoxicity varies a lot from person to person. Age, food intake, genetic variations in APAP-metabolizing enzymes, and concurrent alcohol consumption have all been linked to different clinical outcomes after an APAP overdose. Alterations in gut microbiota have been linked to liver diseases. Diurnal differences in gut microbiota abundance and composition have been linked to APAP-induced hepatotoxicity susceptibility (Christoph et al., 2016). However, the gut microbiota produces thousands of tiny, chemically varied compounds, most of which have unclear biological activities and molecular mechanisms. According to our 16s sequencing data with intestinal contents samples, we found that the percentage of community abundance of *Lachnospiraceae*, *Oscillibacter* and *Colidextribacter* decreased in the OEB pretreatment group compared with that in the APAP group, and that *Akkermansia* and *Parabacteroides* increased on the genus level (Figure 7B). Studies have shown that these gut microbiotas relate to lipid transport and metabolism (Wang et al., 2020; Zhou et al., 2022). *Parabacteroides* alleviates obesity and metabolic dysfunctions via production of succinate and secondary bile acids (Wang et al., 2019)*. Akkermansia* has been found to be lower in several conditions (obesity, diabetes, intestinal inflammation, liver diseases, or chronic alcohol consumption) and this is associated with an altered gut barrier function leading to an increased plasma LPS levels and eventually triggering low grade inflammation and metabolic disorders (Cani and De, 2017). In our data, OEB pretreatment did regulate lipid metabolism (Figure 6). Overall, these observations suggest that OEB regulated lipid metabolism through increasing the abundance of *Akkermansia* and *Parabacteroides*.

## Conclusion

In conclusion, we found the mechanism of hepatoprotective effect of OEB. OEB exerted a potential therapeutic effect against APAP induced hepatotoxicity by inhibiting CYP2E1 and CYP3A11, improving anti-oxidative enzyme activity. Furthermore, OEB decreased APAP hepatotoxicity by regulating lipid metabolism included PPAR signaling. This study indicated that OEB is a potential drug candidate for the hepatoprotection.

## Data Availability

The datasets presented in this study can be found in online repositories. The names of the repository/repositories and accession number(s) can be found below: https://www.ncbi.nlm.nih.gov/, GSE182399.

## Supplementary Material

Supplementary Figure S1 Extraction and separation of active compounds of OEB **(A)** Extract process of *Oplopanax elatus*. **(B)** HPLC profile (UV chromatograms at 208 nm) of OECE, OEA, OEB, and OEC. The main compounds of OEB are chlorogenic acid and isochlorogenic acid A. Chromatography of authentic samples **(C)** chlorogenic acid and **(D)** isochlorogenic acid A.

Supplementary Figure S2 OEB pretreatment affected CYP450 and glucuronosyltransferase and sulfotransferase in vivo. **(A)** the mRNA expression of CYP2E1, **(B)** the mRNA expression of CYP3A11, **(C)** RNA-seq results of drug metabolism heatmap between OEB pretreatment group and APAP group.

Supplementary Figure S3 OEB pretreatment affected PPAR related gene expression in vivo. **(A)** Pparα, **(B)** Fabp1, **(C)** Acaa1b and **(D)** Apoc3.
