# Supplementary figures and images for: Hepatoprotective Effect of Oplopanax elatus Nakai Adventitious Roots Extract by Regulating CYP450 and PPAR Signaling Pathway
Source: Front Pharmacol. 2022 May 2;13:761618. doi: 10.3389/fphar.2022.761618 (PMC9108204; doi:10.3389/fphar.2022.761618)

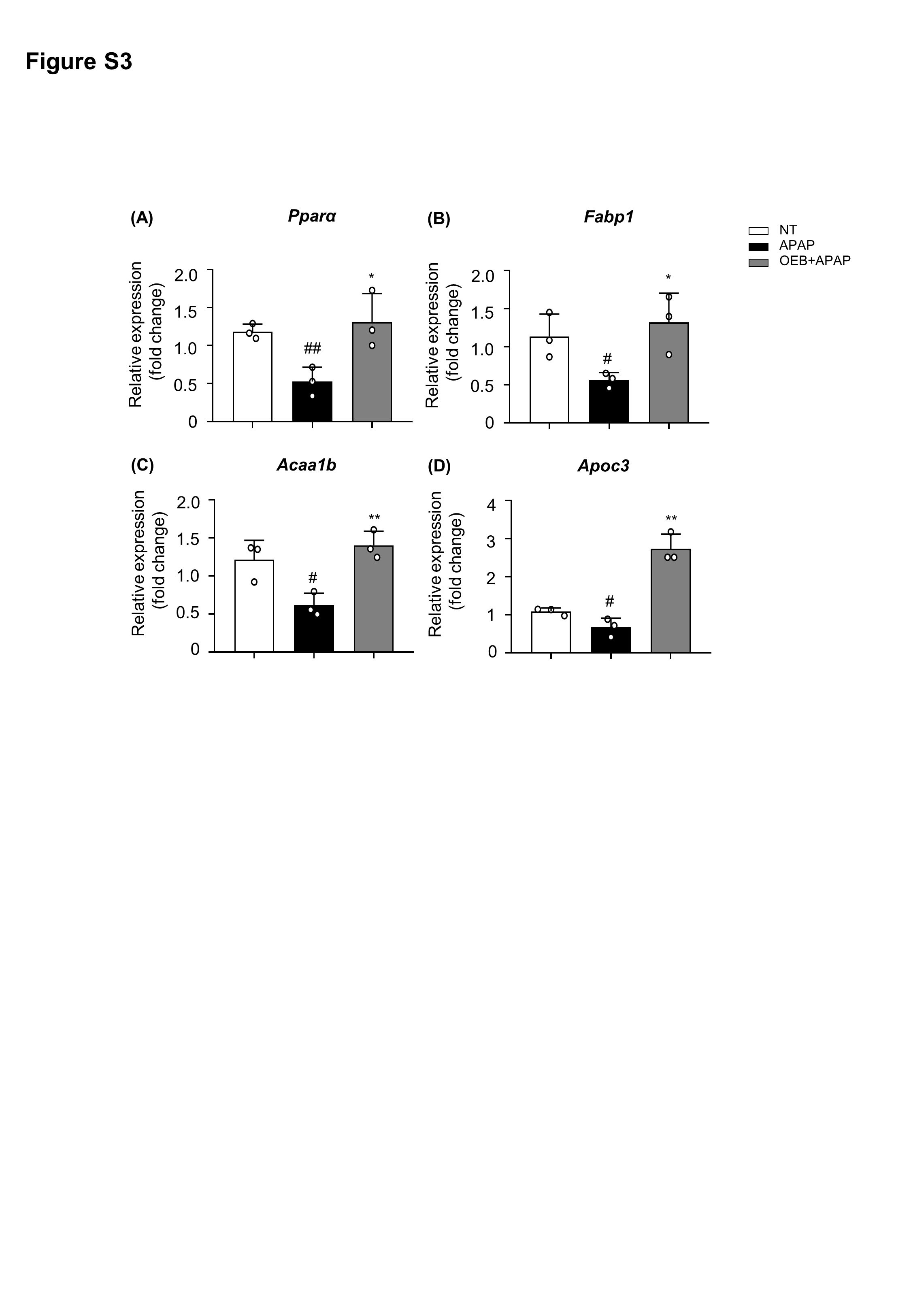

Supplement: Supplementary file 1 [file Image3.TIF]

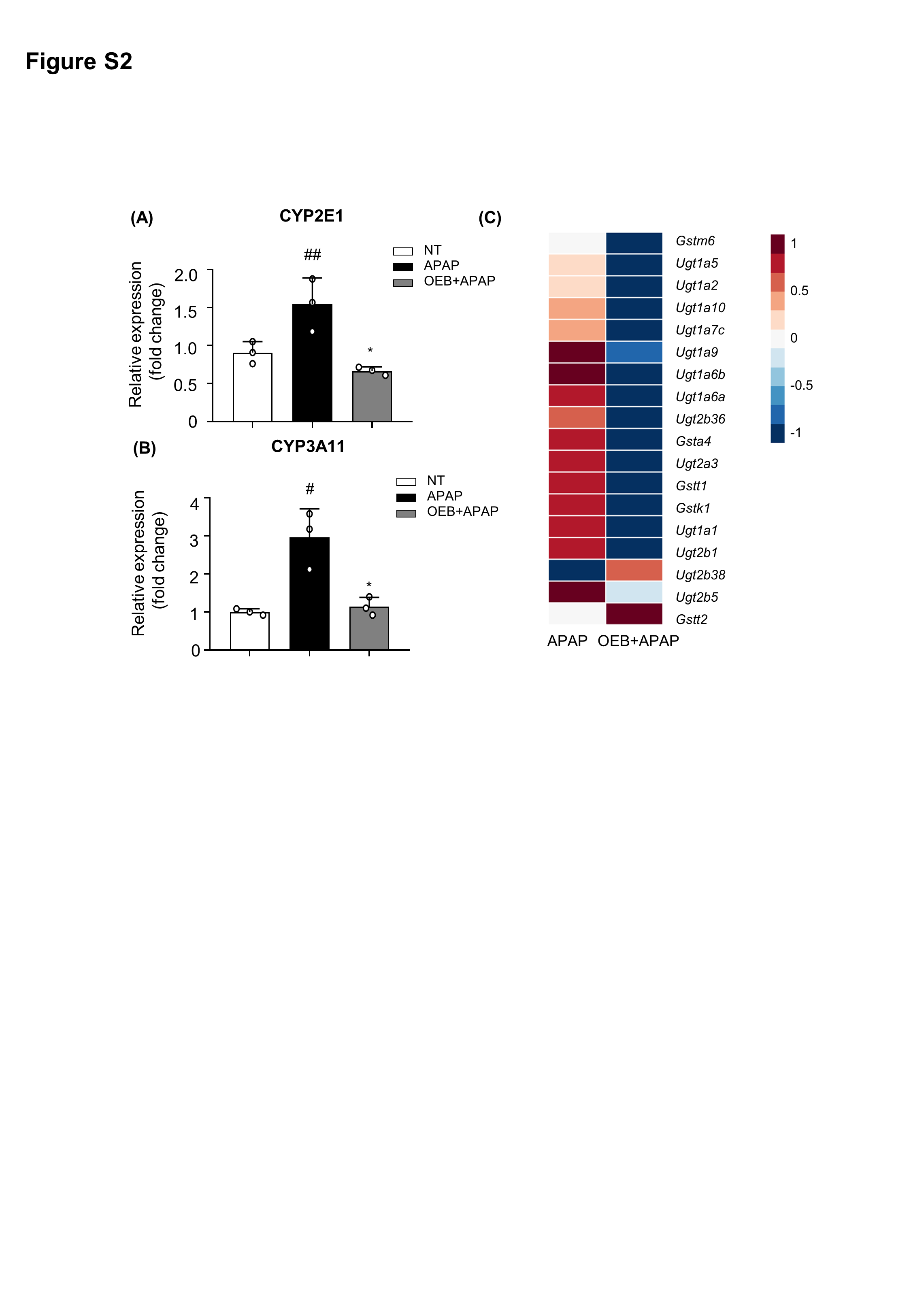

Supplement: Supplementary file 2 [file Image2.TIF]

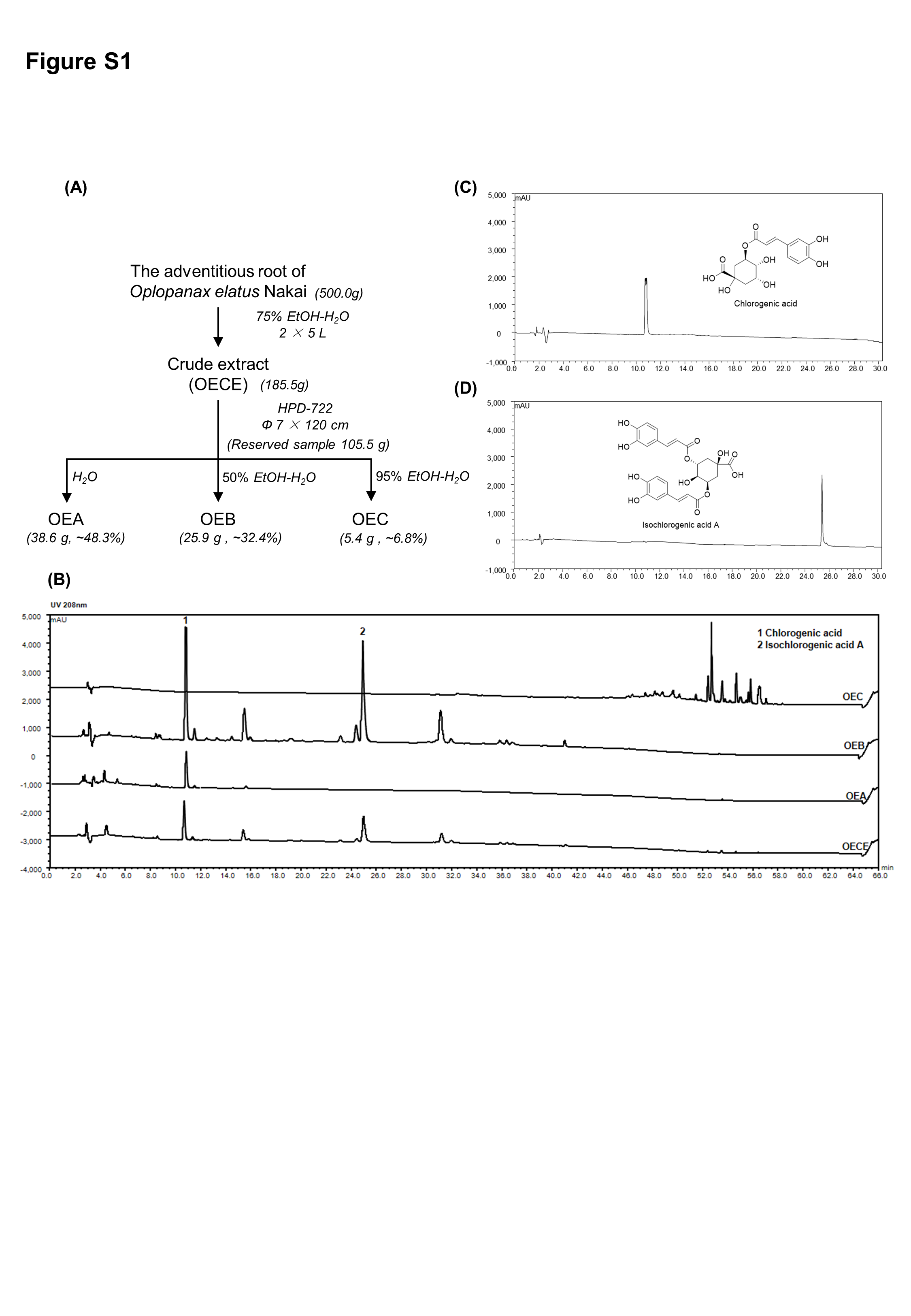

Supplement: Supplementary file 3 [file Image1.TIF]
